# Factors affecting health-related quality of life in patients with skin disease: cross-sectional results from 8,789 patients with 16 skin diseases

**DOI:** 10.1186/s12955-020-01542-6

**Published:** 2020-09-04

**Authors:** Zehui He, Gaetano Marrone, Aihua Ou, Hongxia Liu, Lin Ma, Ying Huang, Yongmei Li, Liyun Sun, Yanping Bai, Wali Liu, Xushan Zha, Chuanjian Lu

**Affiliations:** 1 Department of Clinical Epidemiology, Second Affiliated Hospital of Guangzhou University of Chinese Medicine, Guangzhou, China; 2 Department of Global Public Health, Karolinska Institutet, Stockholm, Sweden; 3 Department of Dermatology, Xinjiang Medical University Affiliated Chinese Medicine Hospital, Urumqi, China; 4 Department of Dermatology, Heilongjiang Academy of Chinese Medical Sciences, Harbin, China; 5 Department of Dermatology, Chengdu University of Traditional Chinese Medicine Affiliated Hospital, Chengdu, China; 6 Department of Dermatology, Shanghai University of Traditional Chinese Medicine Affiliated Longhua Hospital, Shanghai, China; 7 Department of Dermatology, Capital Medical University Affiliated Beijing Traditional Chinese Medicine Hospital, Beijing, China; 8 Department of Dermatology, China-Japan Friendship Hospital, Beijing, China; 9 Department of Dermatology, China Academy of Chinese Medical Sciences Guanganmen Hospital, Beijing, China; 10 Department of Dermatology, First Affiliated Hospital of Guangzhou University of Chinese Medicine, Guangzhou, China; 11 Department of Dermatology, Second Affiliated Hospital of Guangzhou University of Chinese Medicine, No. 111 Da De Road, Yue Xiu District, Guangzhou, 510120 Guangdong China; 12 Guangdong Provincial Key Laboratory of Clinical Research on Traditional Chinese Medicine Syndrome, Guangzhou, China

**Keywords:** Dermatology, Logistic models, Quality of life, Skin diseases

## Abstract

**Background:**

Many previous studies have reported factors that contribute to health-related quality of life (HRQoL) for a single skin disease. However, little is known about generalized factors associated with HRQoL across skin diseases. The objective of this study was to investigate overall HRQoL, and to identify factors related to severely impaired HRQoL among patients with 16 different skin diseases.

**Methods:**

A cross-sectional study of 9845 patients with skin disease was conducted in 9 hospitals in China. HRQoL was assessed with the Chinese version of the Skindex-29 which measures dermatology-specific health along three domains (symptoms, emotions and functioning). With the published Skindex-29 cut-off scores for severely impaired HRQoL, logistic regression models assessed the relationship between severely impaired HRQoL and demographic/clinical characteristics, with adjustments for different skin diseases. To guarantee the models’ convergence, 16 skin diseases with frequencies of at least 100 were included, and the sample size was 8789.

**Results:**

Emotions was the most impaired aspect of HRQoL. Co-existing chronic diseases, 3 years or longer duration, and more severity were identified as associated factors for severely impaired HRQoL for each Skindex-29 domain, and for the aggregate. Being female, under 45 years old, and consuming alcohol were associated with a severely impaired emotion domain; Lack of exercise and smoking were associated with severely impaired symptoms and function domains, respectively.

**Conclusions:**

Skin diseases can affect many facets of HRQoL, but the emotional impairment deserves more attention. In addition to skin disease severity, this study shows that other chronic diseases and long duration are correlated with severely impaired HRQoL for patients with 16 clinical common skin diseases. This suggests the need for increased awareness in treating skin disease as a chronic disease. It also suggests that disease management decisions should consider HRQoL improvement, especially emotional conditions, when making management decisions.

## Introduction

Skin diseases are an enormous global public health burden, with 3 of them among the 10 most prevalent diseases globally [1, 2]. Regarded as a category of chronic disease, most common skin diseases are rarely life-threatening, however, they diminish the quality of life of most individuals who suffer from them. The burden of skin disease encompasses physical, psychological, and social consequences [3]. Accurate measures of the burden of skin disease must assess not only disabilities, but also the effects on patients’ quality of life [4].

The effect of skin disease on health-related quality of life (HRQoL) has been recognized and documented. Many previous studies on psoriasis, acne vulgaris, cutaneous lupus erythematosus, alopecia areata, urticaria, and vitiligo have demonstrated how these skin diseases impair HRQoL [5–11]. Various factors have been identified as being related to the HRQoL of patients with a specific skin condition. For example, age, employment status, marital status, disease duration and self-reported severity have each been determined to be related to the HRQoL of psoriasis patients [5]. Sex, age, generalized disease, distribution of lesions, and severity have been determined to be related to the HRQoL of patients with cutaneous lupus erythematosus [7]. However another study, also on cutaneous lupus erythematosus, determined that sex, education, income, presence of systemic lupus erythematosus, and disease activity were correlated with poor HRQoL [8]. Various factors contribute to the HRQoL of patients with different skin conditions (e.g., psoriasis and cutaneous lupus erythematosus) [5, 7], and even for patients with the same skin condition (e.g., cutaneous lupus erythematosus) [7, 8].

Skin diseases, as a category of chronic disease, have a long-term effects on HRQoL. It is essential to understand skin diseases’ effect and their determinants as a class of chronic disease, and to collect results for the array of common skin conditions. Therefore, the objective of this study is two-fold: to investigate the overall HRQoL of patients with skin diseases; and to identify demographic and clinical factors that affect HRQoL. This was accomplished with logistic regression analysis employing adjustments for different skin diseases.

## Methods

### Sample

This is a non-interventional, cross-sectional study conducted on dermatology outpatients and inpatients with skin diseases. It was approved by the ethics committee at the Guangdong Provincial Hospital of Chinese Medicine.

Patients at least 16 years of age and with confirmed skin disease diagnosis were recruited between May 2013 and December 2015 in 9 Chinese hospitals. Patients were excluded if they suffered from mental illness or were physically unable to complete the survey.

The study was explained to the patients during their visit to either the dermatology outpatient or inpatient clinic by the trained investigators. If patients were willing to participate, written informed consent was obtained. All participants completed their paper questionnaires independently, and then the investigators checked them to ensure every item had been answered.

### Measurement tools

The patients provided socio-demographic data (sex, age, height, weight, education level, marital status, employment, smoking, alcohol consumption, exercise), history of other co-existing chronic diseases (such as hypertension, diabetes, or arthritis), and skin disease duration.

Diagnosis of skin disease was confirmed by at least two dermatologists. Each of the dermatologists had been trained before the formal observations commenced to ensure their diagnoses would follow the same criteria. A dermatologist assessed severity using a global assessment scale with five-point ratings from “slight” to “very severe”. Dermatologists were also provided with the written standard severity assessment documents to ensure that the assessments were consistent. Specific measures for a single skin disease (e.g., the Psoriasis Area or the Severity Index for psoriasis) were not used. This was because one specific measure would not have been applicable to all skin diseases.

HRQoL data were collected using the Chinese version of the dermatology-specific questionnaire. As has been used in a variety of skin conditions [12], Skindex-29 was the HRQoL outcome in this study. Before the cross-sectional study, MAPI Research Trust, the organization holding the copyright for the use of the Skindex29, gave us permission to use it. Evidence suggests that this instrument exhibits good psychometric properties in terms of measuring HRQoL among patients with skin disease, and in Chinese patients [12, 13]. It contains 30 items, of which 29 (with the exception of item 18: side-effects of treatment) are assigned to one of three domains: symptoms, emotions and functioning. Items are scored on a five-point response scale, and within each domain, item scores are transformed to a linear scale ranging from 0 to 100. The domain score is the mean of non-missing item scores in a given domain. If over 25% of the responses are missing from any domain, that domain score is missing. Higher scores indicate lower levels of HRQoL.

### Statistical analysis

In addition to descriptive statistics, regression models were used to relate each Skindex-29 domain, and the aggregate, to sample the characteristics. Provisional inspection of the Skindex-29 data showed they were non-normally distributed. Therefore, logistic regression models were used to compare patients with severely impaired HRQoL to the others. The Skindex-29 cut-off scores corresponding to severely impaired HRQoL were identified and showed to have the highest accuracy [14]. As such, the cut-off scores for severely impaired HRQoL of ≥ 52 points on symptoms, ≥ 39 on emotions, ≥ 37 on functioning, and ≥ 44 on the aggregate score were used in this analysis.

All models were adjusted based on specific skin diseases. In order to guarantee the logistic regression models’ convergence, a rule of thumb was that logistic regression models required a minimum of 10 events per explaining variable [15]. For each skin disease included as an explanatory variable in the model, there were at least 10 patients with severe impairment of HRQoL, if the skin diseases was greater than or equal to 100. Therefore, 16 skin diseases with frequencies of at least 100 were included in the models, and the sample size was 8789.

For each Skindex-29 domain, and for the aggregate, logistic models were first used to relate HRQoL to each demographic and clinical characteristic, while adjusting for skin disease. Then, Factors identified as significant (*P* < 0.05) were included in the multivariate model. The final multivariate model was constructed with the enter procedure while the *P*- value for removing factors was set at *P* > 0.1.

Statistical analysis was conducted in SPSS (v 17.0; SPSS Inc., Chicago, IL, USA).

## Results

### Study population

**Fig. 1:**
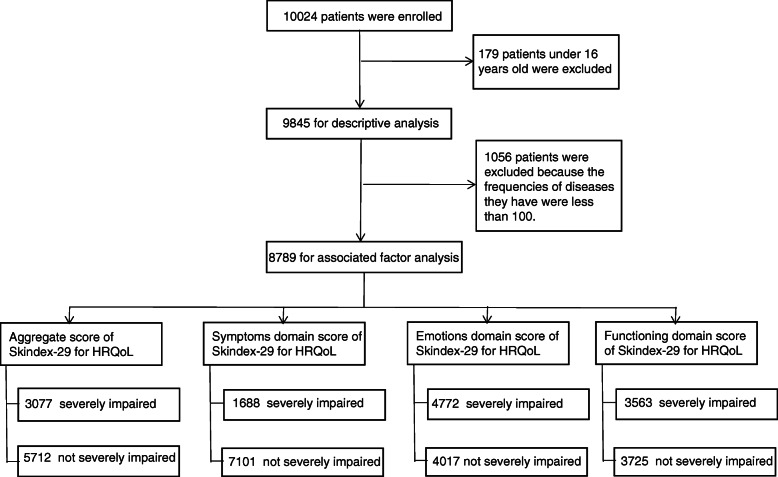
Schematic representation of enrollment and analysis

A total of 10,024 patients were enrolled, with 179 patients under 16 years old excluded from the analysis. This left 9845 patients (98.2%) for descriptive analysis (Fig. 1). Table 1 presents the sample’s summary demographic characteristics. 64% of the participants were female. Participants’ mean age was 33.0 years. Among the participants, 43.4% suffered from other chronic diseases, including respiratory, digestive, and endocrine diseases. The mean duration of their skin disease was 4.0 years (SD 6.0). Half of the participants had moderate disease (50.6%), although 31.8% had mild disease and 13.0% had severe disease.

**Table 1:** Demographic and clinical characteristics of the 9845 patients with skin disease

	Available sample size	
Gender, female (%)	9778	6214 (63.6)
Age, mean (SD)	9845	33.0 (13.5)
Marital status (%)	9715	
Married/living as		4989 (51.4)
Single		4726 (48.6)
Level of education (%)	9760	
Primary school (≤ 6 years)		229 (2.3)
Secondary school (6–9 years)		1074 (11.0)
High school (9–12 years)		2604 (26.7)
College or higher (> 12 years)		5853 (60.0)
Employment (%)	9594	
Employed		5526 (57.6)
Not employed/student		4068 (42.4)
Smoking, yes (%)	9731	3329 (34.2)
Drinking alcohol, yes (%)	9674	3805 (39.3)
Physical exercise, yes (%)	9596	5472 (57.0)
BMI (kg m^−2^), mean (SD)	9514	21.7 (3.3)
Other co-existing chronic diseases yes (%)	9627	4178 (43.4)
Respiratory disease		1077 (11.2)
Digestive diseases		813 (8.4)
Endocrine disease		545 (5.7)
Cardio-cerebrovascular disease		505 (5.2)
Urinary disease		233 (2.4)
Others		440 (4.6)
Missing		565 (5.9)
Duration (years), mean (SD)	8931	4.0 (6.0)
Severity of dermatological disease (%)	9415	
Slight		321 (3.4)
Mild		2995 (31.8)
Moderate		4761 (50.6)
Severe		1220 (13.0)
Very severe		118 (1.3)

*SD* standard deviation, *BMI* body mass index

Overall, 223 skin diseases were collected. They were classified into 21 skin diseases categories. Table 2 presents the frequencies of the 21 skin diseases categories and the 34 skin diseases with frequencies greater than or equal to 10. Acne (29.9%), psoriasis (13.6%), eczema (13.0%), urticaria (7.3%), non-specific dermatitis (5.3%), seborrheic dermatitis (3.2%), alopecia (2.6%), herps (2.6%), contact dermatitis (2.0%), chloasma (1.9%), warts (1.5%), folliculitis (1.4%), neurodermatitis (1.3%), alopecia areata (1.3%), tinea (1.2%), and vitiligo (1.1%) were the 16 diagnoses with frequencies exceeding 100.

**Table 2:** Frequency of categories of skin diseases and dermatological diagnoses under each category

No. of Categories		Frequency	
		Category (%)	Disease (%)
1	Skin appendages diseases	3369 (34.2)	
	**Acne**		2947 (29.9)
	**Alopecia**		260 (2.6)
	**Alopecia areata**		129 (1.3)
	Others		33 (0.3)
2	Dermatitis	2524 (25.6)	
	**Eczema**^a^		1275 (13.0)
	**Non-specific dermatitis**^a^		517 (5.3)
	**Seborrheic dermatitis**		312 (3.2)
	**Contact dermatitis**		200 (2.0)
	**Neurodermatitis**		132 (1.3)
	Atopic dermatitis		57 (0.6)
	Perioral dermatitis		25 (0.3)
	Others		6 (0.1)
3	Erythematous and papulosquamous diseases	1453 (14.8)	
	**Psoriasis**		1335 (13.6)
	Pityiasis rosea		56 (0.6)
	Lichen planus		13 (0.1)
	Others		49 (0.5)
4	**Urticaria** [2]	721 (7.3)	721 (7.3)
5	Viral skin diseases	429 (4.4)	
	**Herps**		259 (2.6)
	**Warts**		151 (1.5)
	Others		19 (0.2)
6	Disturbances of pigmentation	328 (3.3)	
	**Chloasma**		185 (1.9)
	**Vitiligo**		109 (1.1)
	Freckle		10 (0.1)
	Others		24 (0.2)
7	Bacterial infections	169 (1.7)	
	**Folliculitis**		142 (1.4)
	Furuncle		12 (0.1)
	Erysipelas		10 (0.1)
	Others		5 (0.1)
8	Dermatomycoses	159 (1.6)	
	**Tinea**		116 (1.2)
	Onychomycosis		33 (0.3)
	Others		10 (0.1)
9	Cutaneous vasculitis	118 (1.2)	
	Purpura		68 (0.7)
	Erythema nodosum		22 (0.2)
	Vasculitis		10 (0.1)
	Others		18 (0.2)
10	Drug eruptions	78 (0.8)	78 (0.8)
11	Benign skin and vascular tumors	73 (0.7)	
	Melanocytic nevi		29 (0.3)
	Others		44 (0.4)
12	Connective tissue diseases	66 (0.7)	
	SLE		48 (0.5)
	Others		18 (0.2)
13	Pruritic diseases	57 (0.6)	
	Pruritus		32 (0.3)
	Prurigo		25 (0.3)
14	Genital skin diseases	40 (0.4)	
15	Genetic skin diseases	35 (0.4)	
	Ichthyosis		10 (0.1)
	Others		25 (0.3)
16	Vesiculobullous diseases	15 (0.2)	
17	Metabolic skin diseases	13 (0.1)	
	Cutaneous amyloidosis		12 (0.1)
	Others		1 (0.0)
18	Parasitic skin diseases	5 (0.0)	
19	Physical factors induced diseases	4 (0.0)	
20	Skin malignancies	4 (0.0)	
21	Other categories	37 (0.4)	
	Missing	148 (1.5)	
	Total	9845	

^a^More details of this diagnosis were unavailable. Skin diseases with frequency more than 100 were presented in bold

### Skindex-29 scores

Table 3 shows mean scores for the three Skindex-29 domains and the aggregate for both the entire study population and for groups of patients with acne, psoriasis, eczema, urticaria and alopecia.

**Table 3:** Mean scores of Skindex-29 total scale and domains in the study patients and patients with common skin diseases from the literatures

Skindex-29	Total Mean (SD)	Symptoms Mean (SD)	Emotions Mean (SD)	Functioning Mean (SD)	Sample size
Total sample	36.6 (20.9)	34.4 (20.2)	**43.0** (24.8)	32.9 (24.0)	9845
Acne	37.6 (19.3)	33.1 (18.5)	**47.7** (23.9)	32.4 (22.1)	2947
Psoriasis	**44.7** (21.6)	38.7 (19.6)	**51.0** (25.0)	**44.6** (25.8)	1335
Eczema	40.2 (21.6)	42.9 (20.5)	**42.7** (23.9)	35.5 (24.8)	1275
Urticaria	33.4 (18.4)	35.5 (17.6)	35.3 (21.2)	29.8 (21.2)	721
Alopecia	21.2 (16.0)	15.8 (14.6)	29.3 (21.1)	18.5 (18.3)	260
Samples from published studies					
Acne [6]	NA	29.5 (17.9)	34.9 (21.4)	20.3 (20.0)	1878
Psoriasis [16]	33.6 (16.5)	46.4 (18.4)	34.0 (20.2)	20.6 (19.1)	330
Hand eczema [17]	30.3 (17.5)	50.5 (21.1)	32.0 (23.2)	17.2 (16.9)	140
Urticaria [18]	NA	34.7 (8.4)	26.3 (11.2)	23.9 (7.5)	126
Alopecia Areata [9]	NA	12.9 (14.4)	36.2 (25.8)	22.0 (22.6)	60

The cut-off scores for severely impaired HRQoL were 52, 39, 37 and 44 for symptoms, emotions, functioning domain and total score respectively. Values in bold were larger than the cut-off scores for severely impaired HRQoL

*SD* standard deviation, *NA* not available

For the total of 9845 patients with skin disease, the mean of the emotion domain was 43, i.e., over 39. This meant that their emotions had been severely impaired [16]. Additionally, emotion was the most impaired of the three domains. For the patients with acne, psoriasis and eczema, the emotions also reached the severely impaired threshold. In addition, the functioning and aggregate HRQoL score each reached severely impaired for patients with psoriasis.

Skindex-29 scores for patients with acne [6], psoriasis [16], hand eczema [17], urticaria [18] and alopecia areata [9] reported in the literature are also presented in Table 3. In these studies, the symptoms that showed the most impairment, but which did not cross the severely impaired standard, were those for patients with psoriasis, hand eczema and urticaria. The patients in the sample with acne, psoriasis, eczema and urticaria showed worse emotions and functioning than those enrolled in the published studies.

### Factors affecting HRQoL

Based on the statistical analysis mentioned in the methods section, this analysis reported 16 skin diseases with a frequency surpassing 100 (Table 2), and the sample size was 8789 (Fig. 1). There were 3077 (35.0%), 1688 (19.2%), 4772 (54.3%) and 3563 (40.5%) patients who exceeded the Skindex-29 cut-off scores corresponding to severely impaired HRQoL for the aggregate scale, and the symptoms, emotions and functioning domains, respectively.

The variation in HRQoL across Skindex-29 domains based on demographic and clinical characteristics was examined, and each of these factors was analyzed separately by adjusting for skin disease. See the Additional File for detailed results of the univariate logistic models. Factors were not included in the multivariate model if they had no statistically significant association with the severely impaired HRQoL in the univariate models.

In the multivariate logistic model, the factors with a statistically significant effect on severe impairment of HRQoL were measured for each Skindex-29 domain and for the aggregate. They are shown in Table 4. Other chronic disease, duration and severity had statistically significant associations with severely impaired HRQoL for all three domains, and for the aggregate Skindex-29 scale. The odds ratios were all greater than 1, indicating that co-existing other chronic disease, 3 years or more of duration, and more severity were associated with severely impaired HRQoL.

**Table 4:** Multivariate logistic model relating severely impaired health-related quality of life measured by Skindex-29 to demographic and clinical factors simultaneously adjusting 16 skin diseases

	n	Symptoms Odds Ratio (95% CI)	Emotions Odds Ratio (95% CI)	Functioning Odds Ratio (95% CI)	Total scale Odds Ratio (95% CI)
Gender					
Male (reference)	3185	–	1.00	–	1.00
Female	5548	–	**1.53 (1.37,1.71)**	–	**1.39 (1.24,1.57)**
Unknown	56	–	–	–	–
Age (years)					
< 30(reference)	4758	1.00	1.00	1.00	–
30–45	2469	1.07 (0.89,1.29)	0.98 (0.84,1.14)	1.08 (0.96,1.22)	–
45–65	1268	0.85 (0.68,1.07)	**0.58 (0.47,0.71)**	0.89 (0.75,1.05)	–
≥ 65	294	1.25 (0.89,1.75)	**0.65 (0.47,0.89)**	1.15 (0.85,1.56)	–
Marital status					
Married / living as (reference)	4396	1.00	1.00	–	–
Single	4283	0.96 (0.81,1.14)	1.08 (0.93,1.24)	–	–
Unknown	110	–	–	–	–
Education level					
≤9(reference)	1134	–	1.00	1.00	1.00
9–12	2283	–	1.19 (1.00,1.42)	1.01 (0.85,1.20)	1.03 (0.86,1.22)
> 12	5301	–	0.96 (0.82,1.13)	0.88 (0.76,1.03)	0.90 (0.77,1.06)
Unknown	71	–	–	–	–
Employment					
Employed (reference)	4947	–	–	–	–
Not-employed/student	3632	–	–	–	–
Unknown	219	–	–	–	–
Smoke					
No (reference)	5723	–	–	1.00	1.00
Yes	2971	–	–	**1.19 (1.03,1.37)**	**1.22 (1.05,1.42)**
Unknown	95	–	–	–	–
Drink alcohol					
No (reference)	5237	–	1.00	1.00	1.00
Yes	3401	–	**1.23 (1.09,1.38)**	1.00 (0.87,1.14)	1.10 (0.96,1.27)
Unknown	151	–	–	–	–
Exercise					
No (reference)	3677	1.00	–	–	–
Yes	4899	**0.85 (0.75,0.97)**	–	–	–
Unknown	213	–	–	–	–
BMI					
< 25(reference)	7224	–	1.00	–	–
25–30	1114		1.01 (0.86,1.18)	–	–
≥30	151	–	0.88 (0.60,1.29)	–	–
Unknown	300	–	–	–	–
Other chronic disease					
No (reference)	4904	1.00	1.00	1.00	1.00
Yes	3693	**1.37 (1.20,1.56)**	**1.34 (1.20,1.51)**	**1.28 (1.14,1.43)**	**1.24 (1.10,1.40)**
Unknown	192	–	–	–	–
Duration (years)					
< 1(reference)	2694	1.00	1.00	1.00	1.00
1–3	2084	**1.30 (1.10,1.54)**	1.10 (0.96,1.25)	0.99 (0.87,1.13)	1.11 (0.97,1.28)
≥3	3217	**1.47 (1.26,1.71)**	**1.46 (1.29,1.65)**	**1.22 (1.08,1.38)**	**1.39 (1.23,1.58)**
Unknown	794	–	–	–	–
Severity					
Slight (reference)	278	1.00	1.00	1.00	1.00
Mild	2734	**1.47 (0.92,2.37)**	**1.63 (1.20,2.21)**	**1.42 (1.03,1.98)**	**1.76 (1.22,2.54)**
Moderate	4316	**2.75 (1.72,4.38)**	**3.06 (2.27,4.13)**	**2.78 (2.01,3.83)**	**3.40 (2.36,4.89)**
Severe	1082	**4.29 (2.65,6.94)**	**5.96 (4.28,8.29)**	**5.23 (3.70,7.38)**	**6.69 (4.57,9.80)**
Very severe	104	**10.66 (5.66,20.09)**	**14.48 (7.23,29.00)**	**13.27 (7.07,24.94)**	**15.34 (8.28,28.44)**
Unknown	275	–	–	–	–

Odds ratio > 1 indicated lower HRQoL in the corresponding category. Significant results were presented in bold. Dashes indicate inapplicable or not included in the model. *CI* confident interval, *BMI* body mass index

Several demographic factors were significant in some domains. For the symptoms domain, exercise status was statistically significant, with an odds ratio of 0.85 (95% CI: 0.75, 0.97) (exercise compared with no exercise). This indicated that no exercise was associated with symptoms causing severe impairment. For the emotion domain, sex, age and alcohol consumption were insignificant, with odds ratios of 1.53 (female to male), 0.58 (age 45–65 to < 30 years), 0.65 (age ≥ 65 to < 30 years) and 1.23 (alcohol consumption to no alcohol consumption), respectively. This indicated that being female, under 45 years old and drinking alcohol were associated with severely impaired emotions. For the functioning domain, smoking was insignificant, with an odds ratio of 1.19 (smoking to no smoking). This indicated that smoke was associated with severely impaired functioning. For the aggregate scale, being female and smoking were the two factors associated with severely impaired HRQoL.

Neither education level, employment, nor body mass index (BMI) were found to be associated with severely impaired HRQoL.

## Discussion

To our knowledge, this is the most extensive cross-sectional study to investigate factors on a standardized measure of HRQoL among patients with skin disease, with the ability to simultaneously adjust for multiple skin conditions. The key finding was the three factors (other chronic disease, duration and severity) that were associated with severely impaired HRQoL, as measured by each Skindex-29 domain score and the aggregate. Another essential finding was that several demographic variables, such as being female, alcohol consumption, and physical exercise, were also significant.

Regarded as a category of chronic disease, skin diseases have substantial effects on patients’ HRQoL, and especially on the emotion domain. Its average score was higher than the cut-off score for severely impaired (Table 3). This most noticeable effect could have been due to the visibility of most skin disease symptoms, and the influence on daily activities and work associated with emotional distress. Our finding confirmed the importance of considering the psychological impairment of patients with skin diseases. This accords with the results of previous studies [6, 9, 10, 19, 20].

Studies have also consistently concluded that there is increased psychiatric comorbidity and worse HRQoL among female patients with skin disease [6, 21–23], and more emotional impairment at a younger age [7]. Our study corroborated these results, and we found that female patients presented a higher likelihood of having severe impaired emotional and aggregate quality of life. This could be due to the prevalence and severity of some skin diseases in females. Moreover, we also noted that alcohol consumption was associated with severe emotional impairment. Alcoholism has been shown to be a significant factor in suicide, and has often been linked to the emotional disorders that accompany skin disorders [24].

As expected, disease severity contributed to the HRQoL of patients with skin diseases. Similar results have been found in several studies on patients with psoriasis, acne, cutaneous lupus erythematosus and alopecia areata [5–7, 9]. Our results also showed that longer duration and other co-existing chronic disease were correlated with severely impaired Skindex-29 scores. This provides further evidence for the long-term effects of skin diseases as chronic conditions. These findings are consistent with the results of studies on psoriasis [5] and cutaneous lupus erythematosus [7]. However, studies on alopecia areata [9], urticaria [10] and vitiligo [11] have not found statistically significant correlations.

The strengths of this study are that it contains a large number of patients with skin diseases, and that it adjusted for a total of 16 skin diseases to identify factors associated with severely impaired HRQoL. These results can be used to quantify the likelihood of severely impaired HRQoL—controlling other factors—for patients with the common skin diseases included in this study. For example, with the results from the model of the aggregate Skindex-29 scores, and supposing that severity is moderate, the likelihood of severely impaired HRQoL is 3.4 times greater than that of slight severity. The likelihood increases 15.3 times that of slight severity if the severity is assumed to be very severe.

Several limitations of this study should be addressed. Recruitment from hospitals took place in a way that likely led to selection bias. It is possible that patients with severe skin conditions had experienced emotions such as shame or embarrassment to a larger extent than patients with less severe skin conditions. Therefore, they may have been reluctant to participate in the study. Additionally, all of the logistic models were adjusted for 16 skin diseases, of which the frequency was at least 100. This guaranteed the models’ convergence. Thus, the generalizability of the results to other skin diseases might be limited. Furthermore, the Skindex-29 cut-off scores for severely impaired HRQoL used in this study were established on a sample from the Netherlands. Therefore, there may have been bias in its application to Chinese patients.

## Conclusions

This study has indicated that the emotion domain is the most affected facet of patients’ HRQoL. Furthermore, suffering from other chronic disease, long duration and more severity were three generalized factors identified as being associated with severely impaired HRQoL among patients with the 16 most common clinical skin diseases. Skin disease should be managed as a chronic disease by improving HRQoL. This should include not only the symptoms of the disease, but also the patients’ psychiatric conditions.

## Supplementary information

**Additional file 1.** It shows the detailed results of the univariate logistic models regressing severely impaired health-related quality of life, as measured by Skindex-29, on demographic and clinical factors.

## Data Availability

The datasets used in the current study are available from the corresponding author upon reasonable request.

## Abbreviations

HRQoL: Health-related quality of life
