# Supplementary material for: Factors affecting health-related quality of life in patients with skin disease: cross-sectional results from 8,789 patients with 16 skin diseases
Source: Health Qual Life Outcomes. 2020 Sep 4;18:298. doi: 10.1186/s12955-020-01542-6 (PMC7487577; doi:10.1186/s12955-020-01542-6)
Supplement: Supplementary file 1 — Additional file 1. It shows the detailed results of the univariate logistic models regressing severely impaired health-related quality of life, as measured by Skindex-29, on demographic and clinical factors. [file 12955_2020_1542_MOESM1_ESM.docx]

Additional table. Univariate logistic models relating severely impaired health-related quality of life measured by Skindex-29 to demographic and clinical factors

|  | n | Symptoms  Odds Ratio (95% CI) | Emotions  Odds Ratio (95% CI) | Functioning  Odds Ratio (95% CI) | Total scale  Odds Ratio (95% CI) |
| --- | --- | --- | --- | --- | --- |
| Gender |  |  |  |  |  |
| Male(reference) | 3185 | 1.00 | 1.00 | 1.00 | 1.00 |
| Female | 5548 | 1.12(0.99,1.26) | 1.35(1.23,1.48) | 1.03(0.93,1.13) | 1.18(1.07,1.30) |
| Unknown | 56 | - | - | - | - |
| Age(years) |  |  |  |  |  |
| <30(reference) | 4758 | 1.00 | 1.00 | 1.00 | 1.00 |
| 30-45 | 2469 | 1.17(1.02,1.33) | 0.96(0.86,1.07) | 1.13(1.01,1.25) | 1.08(0.96,1.21) |
| 45-65 | 1268 | 1.17(0.99,1.39) | 0.75(0.65,0.87) | 1.13(0.98,1.30) | 1.03(0.89,1.19) |
| $\geq$65 | 294 | 1.77(1.34,2.33) | 0.90(0.69,1.16) | 1.65(1.28,2.14) | 1.25(0.96,1.63) |
| Marital status |  |  |  |  |  |
| Married / living as (reference) | 4396 | 1.00 | 1.00 | 1.00 | 1.00 |
| Single | 4283 | 0.87(0.77,0.98) | 1.11(1.01,1.22) | 0.91(0.83,1.00) | 0.94(0.85,1.03) |
| Unknown | 110 | - | - | - | - |
| Education level |  |  |  |  |  |
| $\leq$9(reference) | 1134 | 1.00 | 1.00 | 1.00 | 1.00 |
| 9-12 | 2283 | 0.89(0.75,1.07) | 1.17(1.01,1.36) | 0.97(0.84,1.13) | 1.00(0.86,1.17) |
| >12 | 5301 | 0.90(0.77,1.06) | 1.01(0.88,1.15) | 0.84(0.73,0.96) | 0.85(0.74,0.98) |
| Unknown | 71 | - | - | - | - |
| Employment |  |  |  |  |  |
| Employed(reference) | 4947 | 1.00 | 1.00 | 1.00 | 1.00 |
| Not-employed/student | 3632 | 0.94(0.84,1.05) | 0.99(0.90,1.08) | 0.93(0.85,1.02) | 0.93(0.85,1.02) |
| Unknown | 219 | - | - | - | - |
| Smoke |  |  |  |  |  |
| No(reference) | 5723 | 1.00 | 1.00 | 1.00 | 1.00 |
| Yes | 2971 | 1.00(0.89,1.12) | 1.08(0.99,1.19) | 1.20(1.09,1.31) | 1.17(1.07,1.29) |
| Unknown | 95 | - | - | - | - |
| Drink alcohol |  |  |  |  |  |
| No(reference) | 5237 | 1.00 | 1.00 | 1.00 | 1.00 |
| Yes | 3401 | 0.95(0.85,1.06) | 1.12(1.03,1.23) | 1.10(1.01,1.20) | 1.11(1.01,1.22) |
| Unknown | 151 | - | - | - | - |
| Exercise |  |  |  |  |  |
| No(reference) | 3677 | 1.00 | 1.00 | 1.00 | 1.00 |
| Yes | 4899 | 0.89(0.80,0.99) | 0.92(0.85,1.01) | 1.02(0.93,1.11) | 0.97(0.88,1.06) |
| Unknown | 213 | - | - | - | - |
| BMI |  |  |  |  |  |
| <25(reference) | 7224 | 1.00 | 1.00 | 1.00 | 1.00 |
| 25-30 | 1114 | 0.99(0.84,1.16) | 0.87(0.76,0.99) | 1.04(0.90,1.19) | 0.97(0.85,1.12) |
| $\geq$30 | 151 | 1.03(0.70,1.54) | 0.87(0.62,1.23) | 1.10(0.79,1.55) | 0.97(0.68,1.37) |
| Unknown | 300 | - | - | - | - |
| Other chronic disease |  |  |  |  |  |
| No(reference) | 4904 | 1.00 | 1.00 | 1.00 | 1.00 |
| Yes | 3693 | 1.29(1.16,1.44) | 1.25(1.14,1.37) | 1.27(1.16,1.39) | 1.30(1.19,1.43) |
| Unknown | 192 | - | - | - | - |
| Duration (years) |  |  |  |  |  |
| <1(reference) | 2694 | 1.00 | 1.00 | 1.00 | 1.00 |
| 1-3 | 2084 | 1.37(1.17,1.60) | 1.08(0.95,1.21) | 1.00(0.88,1.13) | 1.10(0.97,1.25) |
| $\geq$3 | 3217 | 1.67(1.45,1.93) | 1.46(1.30,1.63) | 1.32(1.18,1.47) | 1.47(1.31,1.65) |
| Unknown | 794 | - | - | - | - |
| Severity |  |  |  |  |  |
| Slight(reference) | 278 | 1.00 | 1.00 | 1.00 | 1.00 |
| Mild | 2734 | 1.53(0.99,2.38) | 1.55(1.19,2.02) | 1.43(1.06,1.92) | 1.63(1.17,2.27) |
| Moderate | 4316 | 2.94(1.91,4.52) | 2.76(2.12,3.59) | 2.65(1.98,3.56) | 3.01(2.18,4.16) |
| Severe | 1082 | 4.85(3.10,7.58) | 4.93(3.68,6.59) | 5.16(3.77,7.07) | 5.91(4.20,8.33) |
| Very severe | 104 | 12.32(6.80,22.34) | 10.02(5.48,18.31) | 11.48(6.48,20.33) | 13.36(7.58,23.54) |
| Unknown | 275 | - | - | - | - |

Dashes indicate inapplicable or not included in the model. CI, confident interval; BMI, body mass index
